# Role of p53 isoforms and aggregations in cancer

**DOI:** 10.1097/MD.0000000000003993

**Published:** 2016-07-01

**Authors:** SeJin Kim, Seong Soo A. An

**Affiliations:** Department of Bionano Technology, Gachon University, Gyeonggi-do, Republic of Korea.

**Keywords:** aggregation, cancer, isoform, mutation, p53, protein, tumor

## Abstract

p53 is a master regulatory protein that is involved in diverse cellular metabolic processes such as apoptosis, DNA repair, and cell cycle arrest. The protective function of p53 (in its homotetrameric form) as a tumor suppressor is lost in more than 50% of human cancers.

Despite considerable experimental evidence suggesting the presence of multiple p53 states, it has been difficult to correlate the status of p53 with cancer response to treatments and clinical outcomes, which suggest the importance of complex but essential p53 regulatory pathways.

Recent studies have indicated that the expression pattern of p53 isoforms may play a crucial role in regulating normal and cancer cell fates in response to diverse stresses. The human *TP53* gene encodes at least 12 p53 isoforms, which are produced in normal tissue through alternative initiation of translation, usage of alternative promoters, and alternative splicing. Furthermore, some researchers have suggested that the formation of mutant p53 aggregates may be associated with cancer pathogenesis due to loss-of function (LoF), dominant-negative (DN), and gain-of function (GoF) effects.

As different isoforms or the aggregation state of p53 may influence tumorigenesis, this review aims to examine the correlation of p53 isoforms and aggregation with cancer.

## 1 Introduction

### 1.1 p53 protein

p53 protein, named after its molecular weight, was discovered by David Lane in 1979 as a protein that is bound to the simian virus (SV40) large T antigen. A few years later, p53 was called “the guardian of the genome” due to its ability to inhibit cancer by maintaining various cellular functions.^[1,2]^ p53 exists as a tetrameric nuclear protein that can interact with many diverse proteins or DNA, whose interactions are regulated by post-translational modifications, such as by phosphorylation, acetylation, ubiquitination, neddylation, sumoylation, and methylation.^[3]^ p53 can be induced by diverse signals, such as by DNA damage, nutrient starvation, heat shock, virus infection, pH change, hypoxia, and oncogene activation.^[4]^ In particular, p53, as a transcription factor, is responsible for the induction or inhibition of gene expression by specific binding to p53-responsive elements (p53REs). These interactions usually occur as a tetramer to finely control proteins related to apoptosis, DNA repair, and cell cycle arrest, and serve to block the transformation of normal cells to cancerous cells by maintaining genetic stability.^[5]^ The p53 evidently is known active as a tetrameric form, and it binds with high affinity to DNA or interacts more efficiently with various other proteins in this conformation. The ability of p53 to distinguish various signals allows it to influence delicate and complex regulatory processes.

In humans, p53 is composed of 393 amino acids, which can be classified into 6 domains: the transcription activation domain (TAD) (residues 1–67), which interacts with a variety of proteins and can be further subdivided into TAD I (residues 1–40) and TAD II (residues 41–67); the proline-rich region (residues 67–98), which is conserved in the majority of p53s; the central core domain (residues 98–303), which includes the DNA-binding domain wherein more than 90% of cancer-causing p53 mutations are found in humans; a nuclear localization signal located at residues 303 to 323; and the tetramerization domain, located at residues 323 to 363; and the C-terminal basic domain (residues 363–393), which contains a nonspecific DNA-binding domain that recognizes and binds to damaged DNA.^[6]^

The importance of p53 as a tumor suppressor is clearly evident, considering that more than 50% of all malignant tumors are caused by mutations in the *TP53* gene. Furthermore, more than 90% of TP53 point mutations are located in the central core domain.^[7]^ Inactivation of p53-regulated pathways through point mutations dramatically increases susceptibility to cancer. Cancer can occur even when no p53 mutations are present, but the p53 pathway is disrupted.^[8]^ Many studies were conducted to elucidate the mechanisms underlying the above mentioned phenomena. Recently, numerous researchers have focused on the potential importance of different isoforms and the aggregation states of p53, which we will discuss as follows.

### 1.2 Isoform of p53 protein

#### 1.2.1 The structure and function of p53 isoforms

**Figure 1:**
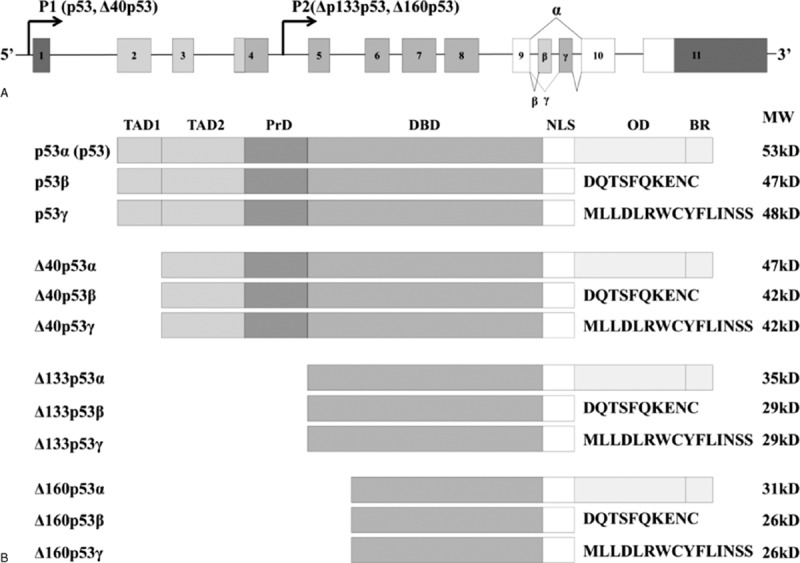
The *TP53* gene can be expressed with 12 different isoform proteins from the alternative initiation of translation, usage of alternative promoters and alternative splicing. (A) Schema of the human *p53* gene structure: alternative splicing sites (α, β, γ) and promoters (P1, P2) are marked. (B) Schema of the human p53 protein isoforms can be expressed by the human p53 gene. BR = basic region, DBD = DNA-binding domain, NLS = nuclear localization signal, OD = oligomerization domain, PRD = proline-rich domain, TAD = transcription activation domain.

The *TP53* gene is composed of 11 exons (Fig. 1A). p53 isoforms were first discovered by Matlashewski in 1984. One year later, Rotter et al investigated alternatively spliced C-terminal variants of mouse p53, and their results were subsequently confirmed in human cells.^[9–11]^ In theory, the *TP53* gene can be expressed as 12 different p53 isoforms (p53α, p53β, p53γ, Δ40p53α, Δ40p53β, Δ40p53γ, Δ133p53α, Δ133p53β, Δ133p53γ, Δ160p53α, Δ160p53β, and Δ160p53γ) through alternative initiation of translation, usage of alternative promoters, and alternative splicing.^[11–13]^Figure 1B shows a schema of the different p53 isoforms encoded by the human *p53* gene. The most abundant p53 isoform, the canonical p53 protein (p53α or p53), has the full TAD sequence and the longest C-terminal domain. In addition, depending on the translation initiation site, 3 ΔN variants, Δ40p53, Δ133p53, Δ160p53, can be expressed. These 4 N-terminal variants can be combined with 3 different C-terminal domains (α, β, γ). p53 isoforms are not only expressed differently for different cancer types but they also have different transcriptional activities and tumor-suppressor functions that can affect various other biological functions. Currently, researchers reported the existence of various isoforms of p53, but their biological functions have not been fully investigated. In short, p53β was reported to enhance the transcriptional activity of p21 through p53(α) pathway and through BCL2-associated X protein (BAX) promoters. p53β can also lead to apoptosis through p53-independent manner.^[14]^ On the contrary, p53γ could enhance the transcriptional activity of only through BAX promoter.^[15]^ Interestingly, Δ40p53α could influence the dominant-negative effect to p53(α), interfering transcriptional activities.^[12]^ In addition, interactions between Δ133p53α and p53(α) could regulate the gene expressions by arresting apoptosis, G1 cell-cycle arrest, and replicative senescence, and enhancing blood vessel formation, metastasis formation, and endothelial cell migration.^[16]^ Hence, the functions of various p53 isoform could strengthen or interfere the tumor suppressor activity. Recently, the expression patterns of p53 isoforms were investigated for their importance in regulating gene expression in cancer cells compared with that in normal cells. Hence, the expression of abnormal p53 isoforms, regardless of the mutation, may contribute to the development of cancer.

#### 1.2.2 p53 isoforms and cancer

p53 plays an important role in the growth and progression of cancer and its response to chemotherapy. Despite many studies, it was difficult to predict these factors with only p53 status. Many institutions have independently performed clinical studies trying to correlate the expression patterns of p53 isoforms, at the mRNA and protein levels, with various types of cancers.^[14,17–25]^ Collectively, results from these clinical studies suggest that specific expression patterns of p53 isoforms may be associated with tumor progression, clinical response, and prognoses. The expression patterns of p53 isoforms were studied in various cell lines, such as colorectal adenoma, renal cell carcinoma, mucinous ovarian cancer, serous ovarian cancer, breast cancer, cholangiocarcinoma, acute myeloid leukemia (AML), squamous cell carcinoma of the head and neck (SCCHN), and melanoma cell lines. Fujita et al^[17]^ showed that the progression of colorectal adenoma to carcinoma could be predicted by the ratio of p53β to Δ133p53α. In renal cell carcinoma, p53β was evaluated as a potential progression marker at both the mRNA and protein levels, and p53β overexpression appeared to be associated with tumor progression.^[18]^ In addition, the expression of Δ40p53α in mucinous ovarian cancer was found to improve recurrence-free survival rates.^[19]^ Δ133p53α and Δ40p53α isoforms were assessed in serous ovarian cancer cases as potential biomarkers.^[20]^ p53α, p53β, and p53γ isoforms are expressed in normal breast cells, but following the expression pattern of the p53γ isoform alone may be sufficient to predict good prognosis.^[21]^ In a subsequent study, the expression patterns of p53 isoforms were analyzed in 30 cases of primary breast tumors. In 18 cases, loss of p53β and p53γ expression was seen,^[14]^ whereas in 12 cases, overexpression of the isoform Δ133p53 was seen.^[14]^ The expression patterns of isoforms in tumors differed from those in normal tissue, and could differ even between each tumor case. The expression of Δ133p53 in cholangiocarcinoma correlated with poor clinical outcomes.^[22]^ In contrast, overexpression of p53β and p53γ was correlated with improved responses to chemotherapy in AML.^[23]^ In SCCHNs, elevated levels of the p53β isoform were found in a large number of samples.^[24]^ In a study on melanoma, elevated levels of mRNA for p53β and Δ40p53 were detected in tumor cells but not in melanocytes or fibroblasts.^[25]^ The expression patterns of p53 isoforms in the various tumors mentioned above are summarized in Table 1.

**Table 1:**
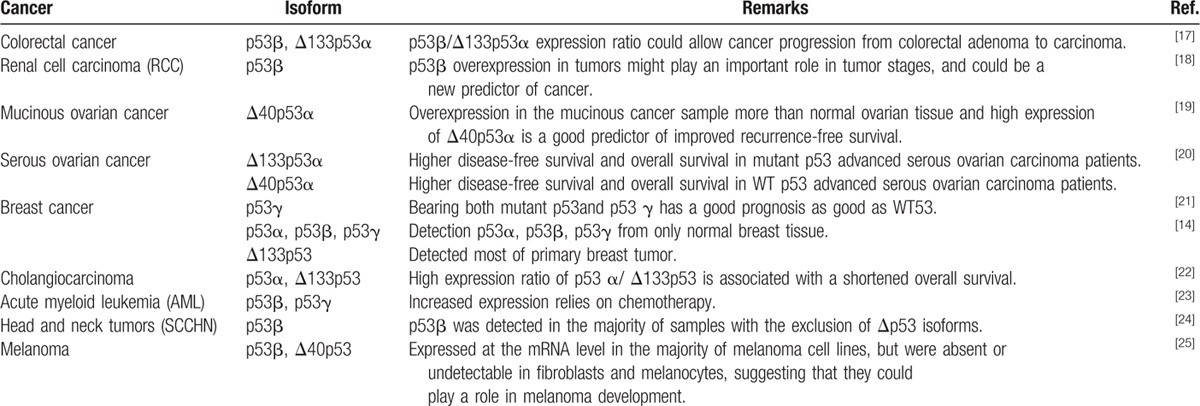
Role of p53 isoforms in human cancer.

p53 isoforms could be found in various cancers and their compositions may vary. These dynamic changes of p53 isoforms may affect the main known function of p53 as tumor suppressor. Hence, understanding and monitoring p53 isoforms at the level of mRNA and changes in transcription landscape will pave the directions of future studies of p53 isoforms. In addition, Khoury et al^[26]^ and Marcel et al^[27]^ suggested to measure the changes in p53 mRNA levels by using nested RT-PCR and quantitative real-time RT-PCR and p53 protein quantification by using western blotting, immunoprecipitation, and luciferase reporter gene assays.

Although diverse p53 isoforms were studied clinically by using various types of cancer models with mRNA and protein expression levels, the challenges in correlating p53 expression patterns with cancer prognosis and treatment outcomes may result from investigating only individual isoforms instead of profiling for all isoforms. Hence, further investigation of various aberrant p53 isoforms may help in understanding cancer development and progression, and may ultimately provide novel targets for more effective cancer therapies and tumor markers.

### 1.3 Aggregations of p53 protein

#### 1.3.1 Aggregation concepts

Protein misfolding and aggregation occur in several human disorders, which are commonly referred to as protein misfolding diseases (PMDs). PMDs are characterized by the formation of protein oligomers, protofibrils, and mature fibrils, which can accumulate intra- or extracellularly in several tissues.^[28,29]^ Over the past 2 decades, many neurodegenerative diseases such as Alzheimer disease (amyloid β, tau), Parkinson disease (α-synuclein), Huntington disease (polyglutamine-huntingtin), familial amyloid polyneuropathy (transthyretin), prion diseases (prion, PrPsc), amyotrophic lateral sclerosis (superoxide dismutase 1), and type II diabetes (islet amyloid polypeptide) have been investigated to understand how misfolding or aggregation of their respective proteins induces pathological features.^[28]^ The fundamental paradigm that explains transmission of these aggregates in their respective diseases was pioneered by Stanley Prusiner^[30]^ in the 1980s and is now referred to as the prion hypothesis. Recent research trends in p53 aggregation are based on the prion hypothesis, which will be discussed as follows.

#### 1.3.2 p53 aggregation and cancer

Recently, interest in p53 aggregation has grown among researchers. In fact, the high-molecular weight form of p53 was already detected in 1991.^[31]^ At that time, aggregated p53 was incorrectly considered to be a quaternary structure, which might be produced in order to prevent rapid degradation. However, recently, research on the p53 aggregation has been actively conducted in the motif obtained prion concept. As noted above, many in vitro aggregation studies focused on the central core domain (hot spot mutation with more than 90% genetic modification), and transactivation and tetramerization domains. Ishimaru et al^[32]^ confirmed the formation of various aggregates, which were due to physical interactions of the central domain of p53 (p53C), by using AFM and thioflavin T binding. Later, similar amyloid aggregates formed by the transactivation and tetramerization domains were reported.^[33–35]^ In particular, the p53 hotspot mutant R248Q appeared to self-aggregate more than wild-type p53.^[36]^ p53 mutant (R248Q) seemed to be located in the hotspot of the protein, influencing self-aggregation. In addition, p53 mutant (R248Q) could be influenced by various environment factors, pH, temperature, and pressure. It was reported that p53 mutant (R248Q) could become a seed for the p53 aggregations, including with wild-type p53. The immunofluorescence colocalization assay of breast tissue from biopsy revealed the deposition of p53 aggregates that such aggregates could alter and decrease the normal function of p53 of tumor suppressor function.^[36]^ Aggregation might be due in part to a decrease in the thermodynamic stability of protein conformations in the mutants, which might compensate for this instability by aggregating. A variety of techniques such as electron microscopy, X-ray diffraction, Fourier-transform infrared (FTIR) spectroscopy, dynamic light scattering (DLS), cell viability assays, and anti-amyloid immunoassays could be used to accurately determine the underlying physical mechanisms.^[36]^

Furthermore, it was shown that p53 aggregates may act as seeds for coaggregation with other p53 family proteins such as p63 and p73.^[37]^ It was also shown that full-length and N-terminally truncated protein (p53C) aggregates could be internalized by cells into the cytosol and induce coaggregation with endogenous p53 protein, further supporting the possibility of the prionoid nature of p53 aggregates.^[38]^ Other in vivo evidence has revealed the accumulation of p53 aggregates, which were detected using an amyloid oligomer specific antibody (A11) or a fibrillar-specific antibody (OC), in paraffin-embedded breast tumor biopsy sections and basal cell carcinoma (BCC) cancer samples.^[36,39,40]^ A summary of the above-mentioned results can be found in Table 2.

**Table 2:**
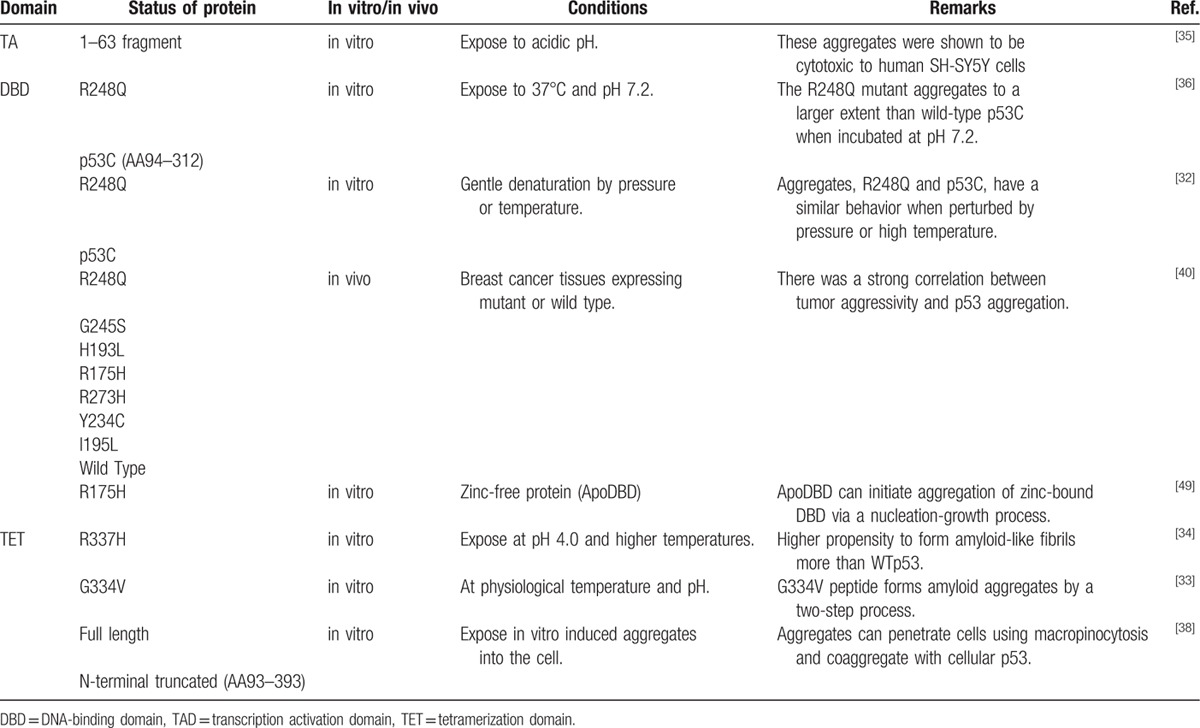
Independent research on p53 aggregation.

Together, these results implicate p53 misfolding and aggregation in cancers and support the hypothesis of prion-like activity of p53 aggregates. As p53 aggregates could coaggregate with wild-type p53, they may have a cytotoxic effect on mammalian cells by hindering the normal functions of p53.^[36]^

### 1.4 Concluding remarks and future perspectives

The tumor-suppressor protein p53 is inarguably important for cancer development and progression and the response to chemotherapy. Despite many studies, it is difficult to predict the therapeutic response and clinical outcome based on p53 alone. Indeed, the p53 pathway can be regulated (i.e., inactivated) in a number of ways. As a result, considerable efforts were made to uncover the mechanisms of inactivation, for example, by p53 protein isoforms and aggregation. Two independent mechanisms have been proposed regarding the isoforms and aggregation of p53. Most of the in vitro methods used to induce p53 aggregation use a partial peptide of the p53C domain, which is similar to the N-terminal truncated p53 isoform (Δ40p53, Δ133p53, and Δ160p53). In addition, robust aggregation of p53 could be induced at low pH, which is thought to simulate the tumor environment.

**Figure 2:**
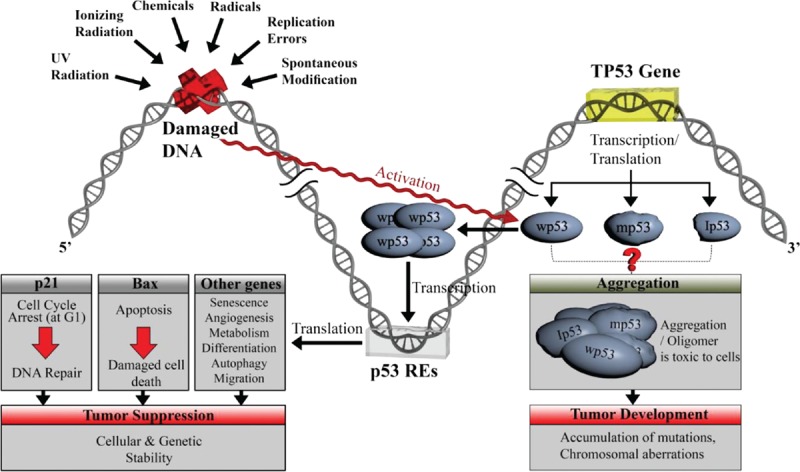
Schematic diagram of cellular responses to DNA damage and hypothesis of p53 aggregate formation. In the normal cellular condition, wp53 tetramer would be formed as one of the cellular stress responses from the DNA damages. p53 as a transcription factor would bind to Res and activate p21, BAX, and other proteins in the tumor suppression pathways. On the contrary, the mutation in the *TP53* gene or other proteins in the downstream pathways in the transcription or translation mechanisms could alter the expressions of mp53 or Ip53, resulting in aggregations of wp53 and suppressing the normal functions of p53. BAX = BCL2-associated X protein, Ip53 = isoform p53, mp53 = mutant p53, p53 REs = p53 response elements, wp53 = wild-type p53.

Furthermore, p53 isoforms were found to form a complex with FLp53 in several studies.^[13,41]^ The formation of this complex could be due to native functional interactions or non-native interactions, which might arise from the formation of aggregates or be due to other causes that inactivate p53. Moreover, in recent studies, the presence of p53 aggregation affected the response of ovarian cancer cells to chemotherapy, which agrees with earlier studies that showed an association between chemoresistance and the presence of shorter p53 isoforms.^[23,42]^ These results strongly suggest a more robust relationship between p53 isoforms and aggregation. In Fig. 2, we propose the following hypothesis to explain how p53 isoforms may induce aggregation. In the normal cellular condition, wp53 tetramer would be formed as one of the cellular stress responsers from the DNA damages. p53 as a transcription factor would bind to REs and activate p21, BAX, and other proteins in the tumor suppression pathways. On the contrary, the mutation in the *TP53* gene or other proteins in the downstream pathways in the transcription or tranalation mechanisms could alter the expressions of mp53 or Ip53, resulting aggregations of wp53 and suppressing the normal functions of p53.

Breaking p53 aggregations similar with prion-like behavior could be the novel therapeutic target in the anti-cancer therapy, as well as other PMDs from protein aggregations, such as Alzheimer disease and Parkinson disease.^[43–48]^

We also propose that further investigations are required to determine the relationship between p53 isoforms and aggregation, especially to clarify their contributions to the regulation of the normal functions of p53. These studies could provide a comprehensive understanding of p53 regulation and functions, and could contribute to the future development of cancer biomarkers and pharmaceutical therapies.
